# A Qualitative Systematic Review About Children’s Everyday Lives when a Parent Is Seriously Ill with the Prospect of Imminent Death - Perspectives of Children and Parents

**DOI:** 10.1177/00302228221149767

**Published:** 2023-01-11

**Authors:** Cong Fu, Stinne Glasdam, Sigrid Stjernswärd, Hongxuan Xu

**Affiliations:** 1 Integrative Health Research, Department of Health Sciences, Faculty of Medicine, 59568 Lund University, Lund, Sweden; 2 Health-promoting Complex Interventions, Department of Health Sciences, Faculty of Medicine, 5193 Lund University, Lund, Sweden

**Keywords:** children, everyday life, interactions, patents’ life-limiting critical illness, qualitative literature review, strategies

## Abstract

Parental dying is a life changing experience for children. This study explores children’s strategies and (inter)actions in their everyday life when facing critically ill parents and imminent death, from the children’s and parents’ perspectives. A qualitative systematic review was carried out, registered in PROSPERO (CRD42022306862). A literature search and screening in six databases resulted in eighteen articles. Thematic analysis showed that children were capable of developing various strategies to cope in everyday life, even in vulnerable situations. From parents’ and children’s perspectives, inclusion, openness, and communication about parents’ situations, taking children’s age and needs into consideration, were important to face and cope with the situation. Children were life-capable, also in vulnerable and difficult situations. This calls for the necessity of developing children-led support, by acknowledging, and taking the children’s experiences, and resources, as starting points to tailor adequate support for children of critically ill parents.

## Introduction

Having a seriously ill parent with the prospect of imminent death strongly marks and affects the everyday life of children and their families (McLaughlin et al., 2019). The prevalence of bereaved children varies across countries. It is, for example, estimated that 2% of children have a deceased parent before age 17 in Sweden (Statistics Sweden, 2018), and children who lost a parent before age 16 were 5% in Great Britain (Childhood Bereavement Network, 2016). A parent dying is regarded as one of the most life changing experiences for children (Guzzo & Gobbi, 2021), and children’s vulnerability in terms of physical health and emotional well-being increases when dealing with drastic life changes (McLaughlin et al., 2019). Previous reviews show that parental death is associated with an increase in depressive episodes (Berg et al., 2016; Pham et al., 2018), and an increased incidence of post-traumatic symptoms and functional impairments among children and young adolescents during the early bereavement period (Pham et al., 2018). Even children with a parent with non-terminal cancer can experience emotional challenges and lack of emotional support in relation to their situation (Huang et al., 2014). Although most studies report negative outcomes in the short and long term on bereaved children after parental death, Walczak and colleagues (2018) found that children also experience positive traumatic growth, such as reinforced family relationships, an increased sense of cherishing life, and increased interest in one’s own health, after a parent’s death. Children of divorced parents seem particularly affected as they are in a double bereavement situation and have experienced loss both through divorce and death. Loss of attachment to important relations and/or to one’s home, and facing a new custodial parent or foster family can significantly affect children (Bugge et al., 2009; Davey et al., 2003; Marcussen et al., 2019; Werner-Lin et al., 2010).

Many earlier review studies reported different types of existing interventions and general positive effects of these intervention for supporting bereaved children across different age groups and children’s caregivers after the death of a parent (Bergman et al., 2017; Chen & Panebianco, 2018; Ellis et al., 2017; Inhestern et al., 2016; Millar et al., 2020; Ridley & Frache, 2020). Review studies also reported children’s need for age-appropriate communication about their parent’s illness and forthcoming death (Ellis et al., 2017) and their needs for flexible and tailored support from family and school (Chen & Panebianco, 2018; Ellis et al., 2017). Several studies showed both healthy parents and ill parents try to maintain a usual everyday life for their children, yet also found it challenging to balance between supporting their children and struggling with their own emotions (Aamotsmo & Bugge, 2014; Semple and Mccance, 2010). Parents with advanced cancer struggled to balance their roles as being a parent and a patient (Matuszczak-Swigon & Bakiera, 2021). The importance of family unity and cohesiveness was significant for parents who have cancer (Matuszczak-Swigon & Bakiera, 2021), for both children and the remaining parent during grief processes (Bylund-Grenklo et al., 2021).

Moreover, families facing parental cancer reported lack of time and collaboration within healthcare services (Inhestern et al., 2016). Healthcare professionals are often reluctant to communicate about the illness both with the parent who has a life-limiting illness and his/her dependent child due to personal emotional challenges, organisational challenges such as lack of time, and perceptions of professional competence and roles (Beverley et al., 2021; Franklin et al., 2019; Karidar et al., 2016; Karidar & Glasdam, 2018). Professional’s skills and attitudes can significantly affect the support to children of ill parents (Beverley et al., 2021), concerning multiple healthcare professionals such as nurses, physicians, and social workers (Karidar et al., 2016; Karidar & Glasdam, 2018). The structural framework, to which healthcare professionals are subject in their work, gives professionals minimal opportunities to find time and space to support relatives to inpatients and outpatients (Ferguson, 2014; 2017; Glasdam & Oute, 2018). Through decades, healthcare professionals and voluntary organisations organise support programmes for bereaved children. However, only few children make use of such offers after a parents’ death (Rosner et al., 2010).

Many children spend a large part of the day at school or kindergarten, where school or kindergarten professionals play a crucial part in supporting children when a parent is critically ill (Duncan, 2020). Hogstad and Jansen (2021) point out that healthcare professionals often focus on and explain biomedical processes of death and dying in simple and concrete ways to minor children, while kindergarten teachers focus on parental death mainly as an emotional phenomenon and include peers of bereaved children in the support strategies. Bereaved children’s needs were often overlooked, although school teachers were aware of the role of school in supporting children after their parent’s death (Dyregrov et al., 2013). This is possibly due to the emotional distress in facing children in grief, insufficient skills and training in death education, and concerns when supporting bereaved children as it is experienced as beyond their professional roles (Lane et al., 2014; Levkovich & Elyoseph, 2021). The integration of death education into school curriculum is limited because of social taboos on death-related topics for both teachers and parents (Friesen et al., 2020; Herrero et al., 2022). Actually, some bereaved children find the school supportive and well-prepared for their changed life situation (Holmgren, 2022), and other children experienced various difficulties with limited support from school teachers as well as peers (Lytje, 2018).

The concept of an everyday life can be seen as both the organisation of everyday tasks and the experience of investigating and meaning-making of these ordinary activities (Ulvik & Gulbrandsen, 2015). Children are social actors and agents in their own daily lives, and they participate in their everyday lives through continually living and interacting with families, schools, communities and their own culture (Hogstad & Leer-Salvesen, 2020). When experiencing a parent’s terminal illness, children might need to develop various methods to cope or negotiate with their everyday life. Yet a professional’s practice with children often relies on the professional’s own knowledge and interests in specific parts of children’s lives, and children’s input is often overlooked (Ulvik & Gulbrandsen, 2015). Life with a critically ill parent may impose burdensome challenges on children in their daily, educational, and social lives (Bennett et al., 2017). A British study further indicated that children’s daily experience of living with a seriously ill parent are shaped by factors such as availability of social services and institutional support (Payler, 2020). Children may be involved in reversed family roles, where they take on caring responsibilities and household work in daily life (Marshall et al., 2021; Payler, 2020). There was a tension between children’s wish to exert their agency and parents' attempt to hide the illness from the children (Marshall et al., 2021), though some studies show that many children wish to be informed or talk about a parent’s impending death (Bylund-Grenklo et al., 2015; Fearnley & Boland, 2017).

The knowledge of how children handle different situations and live their everyday life when a parent is critically ill during the course of their parents’ illness can enhance our understanding of children’s perspective. This study aims to explore children’s strategies and (inter)actions in their everyday life when facing the imminent death of critically ill parents from children’s and parent’s perspectives.

## Method

This study was based on a qualitative systematic literature review synthesising findings across qualitative studies (Bettany-Saltikov & McSherry, 2016) about children’s everyday lives when a parent is seriously ill and dying, from the perspectives of the children and their parents, and through a Braun and Clarke (2006) inspired thematic analysis. This article followed the Preferred Reporting Items for Systematic Reviews and Meta-Analyses (PRISMA) recommendations (Page et al., 2021). The review protocol is registered in PROSPERO (CRD42022306862).

### Identifying Focused Review Question

How do children cope and (inter)act in their everyday life within families, institutions, and communities when living with a parent who is critically ill and dying?

### Search Strategy

A search was conducted in five electronic databases: CINAHL, Embase, Eric, MEDLINE, and PsycINFO, with a building block search carried out according to the population, exposure and outcome (PEO) model (Bettany-Saltikov & McSherry, 2016). The search terms were also consulted with a university librarian. Table 1 presented an example of search terms that were customised in one database. Table 2 presented the full electronic search strategy in each database. These searches were supplemented with a citation pearl search in the Scopus abstract and citation database of the included articles, which were not found in the primary searches.

**Table 1.** Population, exposure and outcome (PEO).

PEO		Search Terms (Examples from Embase)	Search hits
Population Children <18 years old and children’s parents	#1	‘Juvenile’ OR ‘juvenile'/exp OR juvenile OR ‘minor’ OR ‘minor'/exp OR minor OR ‘adolescent’ OR ‘adolescent'/exp OR adolescent OR ‘teenager’ OR ‘teenager'/exp OR teenager OR child* OR ‘dependent child’ OR ‘youth’ OR ‘youth'/exp OR youth OR ‘child’ OR ‘child'/exp OR child OR ‘child preschool'/exp OR ‘child preschool’ OR ‘pre school child'/exp OR ‘pre school child'	5,556,010
	#2	‘sibling'/exp OR sibling OR ‘brother'/exp OR brother OR ‘sister'/exp OR sister	114,772
	#3	#1 OR #2	5,623,273
Exposure Live with a parent who is critically ill and dying	#4	‘Deprivation parental'/exp OR ‘deprivation parental’ OR ‘deprivation paternal'/exp OR ‘deprivation paternal’ OR ‘parent loss'/exp OR ‘parent loss' OR ‘parent deprivation'/exp OR ‘parent deprivation’ OR ‘parental loss'/exp OR ‘parental loss' OR ‘paternal deprivation'/exp OR ‘paternal deprivation’ OR ‘dying parent’ OR ‘dying parents' OR ‘parent dying’ OR ‘maternal deprivation'/exp OR ‘maternal deprivation’ OR ‘imminent dying parent'	5745
	#5	Death NEAR/5 parent*	2934
	#6	Dying NEAR/5 parent*	220
	#7	#4 AND #5 AND #6	8475
Outcomes Children’s coping strategies and (inter)action in their everyday life, and children’s and parents’ perspectives on these coping strategies and (inter)actions	#8	‘Family life'/exp OR ‘family life’ OR ‘daily life'/exp OR ‘daily life’ OR ‘family routine’ OR ‘family functioning'/exp OR ‘family functioning’ OR ‘family function’ OR ‘everyday life’ OR ‘school life’ OR ‘daily experience’ OR ‘school time’ OR ‘social time’ OR ‘spare time’ OR ‘leisure’ OR ‘leisure'/exp OR leisure OR socializing OR ‘social activity'/exp OR ‘social activity’ OR ‘life change events'/exp OR ‘life change events' OR ‘life change event’ OR ‘leisure activities'/exp OR ‘leisure activities' OR ‘adaptation psychological'/exp OR ‘adaptation psychological’ OR ‘coping strateg*' OR ‘activity, daily living'/exp OR ‘activity, daily living’ OR adl OR ‘activities of daily living'/exp OR ‘activities of daily living’ OR ‘daily living activity'/exp OR ‘daily living activity’ OR 'hobbies' OR 'hobbies'/exp OR hobbies OR ‘hobby’ OR ‘hobby'/exp OR hobby OR 'holidays' OR 'holidays'/exp OR holidays OR ‘leisure activity'/exp OR ‘leisure activity’ OR ‘leisure time'/exp OR ‘leisure time’ OR ‘relaxation’ OR ‘relaxation'/exp OR relaxation OR ‘vacation’ OR ‘vacation'/exp OR vacation OR ‘childhood adversity'/exp OR ‘childhood adversity’ OR ‘adverse childhood event'/exp OR ‘adverse childhood event’ OR ‘adverse childhood experience'/exp OR ‘adverse childhood experience’ OR ‘adverse childhood experiences'/exp OR ‘adverse childhood experiences' OR ‘adverse experiences in childhood'/exp OR ‘adverse experiences in childhood’ OR ‘experiences in early childhood’ OR ‘experiences in infancy’ OR ‘child adverse experiences'/exp OR ‘child adverse experiences' OR ‘childhood adverse experience'/exp OR ‘childhood adverse experience’ OR ‘childhood adversities'/exp OR ‘childhood adversities' OR ‘deleterious childhood experiences'/exp OR ‘deleterious childhood experiences' OR ‘physiologic adaptation'/exp OR ‘physiologic adaptation’ OR ‘physiological adjustment'/exp OR ‘physiological adjustment’ OR ‘behavior coping'/exp OR ‘behavior coping’ OR ‘coping ability'/exp OR ‘coping ability’ OR ‘coping behavior'/exp OR ‘coping behavior’ OR ‘coping mechanism'/exp OR ‘coping mechanism’ OR ‘coping strategy'/exp OR ‘coping strategy'	2,453,903
	#9	#3 AND #8	647,320
	#10	#9 AND #7	4974
	#11	#10 AND (2012:py OR 2013:py OR 2014:py OR 2015:py OR 2016:py OR 2017:py OR 2018:py OR 2019:py OR 2020:py OR 2021:py OR 2022:py)	2255

**Table 2.** Full search strings from five databases.

Search From Embase			
PEO		Search Terms (From Embase)	Search hits
Population Children <18 years old and children’s parents	#1	‘Juvenile’ OR ‘juvenile'/exp OR juvenile OR ‘minor’ OR ‘minor'/exp OR minor OR ‘adolescent’ OR ‘adolescent'/exp OR adolescent OR ‘teenager’ OR ‘teenager'/exp OR teenager OR child* OR ‘dependent child’ OR ‘youth’ OR ‘youth'/exp OR youth OR ‘child’ OR ‘child'/exp OR child OR ‘child preschool'/exp OR ‘child preschool’ OR ‘pre school child'/exp OR ‘pre school child'	5,556,010
	#2	‘sibling'/exp OR sibling OR ‘brother'/exp OR brother OR ‘sister'/exp OR sister	114,772
	#3	#1 OR #2	5,623,273
Exposure Live with a parent who is critically ill and dying	#4	‘Deprivation parental'/exp OR ‘deprivation parental’ OR ‘deprivation paternal'/exp OR ‘deprivation paternal’ OR ‘parent loss'/exp OR ‘parent loss' OR ‘parent deprivation'/exp OR ‘parent deprivation’ OR ‘parental loss'/exp OR ‘parental loss' OR ‘paternal deprivation'/exp OR ‘paternal deprivation’ OR ‘dying parent’ OR ‘dying parents' OR ‘parent dying’ OR ‘maternal deprivation'/exp OR ‘maternal deprivation’ OR ‘imminent dying parent'	5745
	#5	Death NEAR/5 parent*	2934
	#6	Dying NEAR/5 parent*	220
	#7	#4 OR #5 OR #6	8475
Outcomes Children’s strategies and (inter)action in their everyday life, and children’s and parents’ perspectives on these coping strategies and (inter)actions	#8	‘Family life'/exp OR ‘family life’ OR ‘daily life'/exp OR ‘daily life’ OR ‘family routine’ OR ‘family functioning'/exp OR ‘family functioning’ OR ‘family function’ OR ‘everyday life’ OR ‘school life’ OR ‘daily experience’ OR ‘school time’ OR ‘social time’ OR ‘spare time’ OR ‘leisure’ OR ‘leisure'/exp OR leisure OR socializing OR ‘social activity'/exp OR ‘social activity’ OR ‘life change events'/exp OR ‘life change events' OR ‘life change event’ OR ‘leisure activities'/exp OR ‘leisure activities' OR ‘adaptation psychological'/exp OR ‘adaptation psychological’ OR ‘coping strateg*' OR ‘activity, daily living'/exp OR ‘activity, daily living’ OR adl OR ‘activities of daily living'/exp OR ‘activities of daily living’ OR ‘daily living activity'/exp OR ‘daily living activity’ OR 'hobbies' OR 'hobbies'/exp OR hobbies OR ‘hobby’ OR ‘hobby'/exp OR hobby OR 'holidays' OR 'holidays'/exp OR holidays OR ‘leisure activity'/exp OR ‘leisure activity’ OR ‘leisure time'/exp OR ‘leisure time’ OR ‘relaxation’ OR ‘relaxation'/exp OR relaxation OR ‘vacation’ OR ‘vacation'/exp OR vacation OR ‘childhood adversity'/exp OR ‘childhood adversity’ OR ‘adverse childhood event'/exp OR ‘adverse childhood event’ OR ‘adverse childhood experience'/exp OR ‘adverse childhood experience’ OR ‘adverse childhood experiences'/exp OR ‘adverse childhood experiences' OR ‘adverse experiences in childhood'/exp OR ‘adverse experiences in childhood’ OR ‘experiences in early childhood’ OR ‘experiences in infancy’ OR ‘child adverse experiences'/exp OR ‘child adverse experiences' OR ‘childhood adverse experience'/exp OR ‘childhood adverse experience’ OR ‘childhood adversities'/exp OR ‘childhood adversities' OR ‘deleterious childhood experiences'/exp OR ‘deleterious childhood experiences' OR ‘physiologic adaptation'/exp OR ‘physiologic adaptation’ OR ‘physiological adjustment'/exp OR ‘physiological adjustment’ OR ‘behavior coping'/exp OR ‘behavior coping’ OR ‘coping ability'/exp OR ‘coping ability’ OR ‘coping behavior'/exp OR ‘coping behavior’ OR ‘coping mechanism'/exp OR ‘coping mechanism’ OR ‘coping strategy'/exp OR ‘coping strategy'	2,453,903
	#9	#3 AND #8	647,320
	#10	#7 AND #9	4974
	#11	#10 AND (2012:py OR 2013:py OR 2014:py OR 2015:py OR 2016:py OR 2017:py OR 2018:py OR 2019:py OR 2020:py OR 2021:py OR 2022:py)	2255

Search from CINAHL			
PEO		Search Terms (From CINAHL)	Search hits
Population Children <18 years old and children’s parents	#1	Juvenile OR juveniles OR minor OR minors OR adolescent OR adolescents OR teenager OR Child*OR “dependent child” OR youth OR “child preschool” OR “pre school child” OR siblings OR brother OR brothers OR sister OR sisters or children	465,582
	#2	(MH “Adolescence+”)	575,807
	#3	(MH “Child+”)	725,200
	#4	(MH “siblings”)	6394
	#5	#1 OR #2 OR #3 OR #4	1,145,403
Exposure Live with a parent who is critically ill and dying	#6	“Deprivation parental “OR “deprivation paternal “OR “parent loss” OR “parent deprivation” OR “parental loss” OR “paternal deprivation” OR “dying parent” OR “dying parents” OR moribund OR “parent dying” OR “maternal deprivation” OR “imminent dying parent” OR “parental death” OR “paternal deprivation “OR “maternal deprivation” OR “parental deprivation” OR “prior to parent death” OR “attitude to parent death” OR “loss of parent*”or “death of parent*”or “parent bereavement” or death or dying or “paternal deprivation” or “maternal deprivation”	732
	#7	(MH “parental death”)	205
	#8	(MH “terminally Ill Patients+”)	12,518
	#9	#6 OR #7 OR #8	13,235
Outcomes Children’s strategies and (inter)action in their everyday life, and children’s and parents’ perspectives on these coping strategies and (inter)actions	#10	MH “family coping” or MH “life Experiences+” OR MH “Death+” OR MH “catastrophic illness” OR MH “family coping” OR MH “family Functioning+” OR MH “family involvement (Iowa NIC)” OR MH “family Relations+”	141,761
	#11	(MH “life change Events+”)	55,897
	#12	(MH “adaptation, Physiological+”) OR (MH “Coping+”)	48,927
	#13	(MH “coaches, athletic”)	1994
	#14	#10 OR #11 #12 #13	215,882
	#15	“Family life” OR “daily life” OR “family routine” OR “family functioning” OR “family function” OR “everyday life”OR “school life”OR “daily experience” OR “school time” OR “social time” OR “spare time” OR leisure OR socializing OR “social activity” OR “adverse childhood experience” OR “life change events “OR “life change event” OR “leisure activities” OR “adaptation psychological” OR “coping strateg*” OR “activities of daily living” OR “activity, daily living” OR ADL OR “daily living activity” OR hobbies OR hobby OR holidays OR “leisure activity” OR “leisure time” OR relaxation OR vacation OR “childhood adversity” OR “adverse childhood event” OR “adverse childhood experiences” OR “adverse experiences in childhood” OR “experiences in early childhood” OR “experiences in infancy” OR “child adverse experiences” OR “childhood adverse experience” OR “childhood adversities” OR “deleterious childhood experiences” OR “physiologic adaptation” OR “physiological adjustment” OR “coping behavior” OR “behavior coping” OR “coping ability” OR “coping behavior ” OR “coping mechanism” OR “coping strategy” OR “daily interaction” OR “family interaction” OR “interaction relationships” OR teachers OR teacher OR coach OR coaches OR “step parents” OR facilitator OR facilitators OR educator OR educators or “school teacher”	279,832
	#16	#14 OR #15	443,877
	#17	#5 AND #16	124,295
	#18	#9 AND #17	653
	#19	Limiters - published date: 2,0120101-20,221,231	380

Search from PsycInfo			
PEO		Search Terms (From PsycInfo)	Search hits
Population Children <18 years old and children’s parents	#1	Juvenile OR juveniles OR minor OR minors OR adolescent OR adolescents OR teenager OR Child*OR “dependent child” OR youth OR “child preschool” OR “pre school child” OR siblings OR brother OR brothers OR sister OR sisters	692,045
	#2	(DE “siblings” OR DE “brothers” OR DE “multiple births” OR DE “sisters”) OR (DE “child behavior”)	17,905
	#3	#1 OR #2	694,545
Exposure Live with a parent who is critically ill and dying	#4	‘Deprivation parental'/exp OR ‘deprivation parental’ OR ‘deprivation paternal'/exp OR ‘deprivation paternal’ OR ‘parent loss'/exp OR ‘parent loss' OR ‘parent deprivation'/exp OR ‘parent deprivation’ OR ‘parental loss'/exp OR ‘parental loss' OR ‘paternal deprivation'/exp OR ‘paternal deprivation’ OR ‘dying parent’ OR ‘dying parents' OR ‘parent dying’ OR ‘maternal deprivation'/exp OR ‘maternal deprivation’ OR ‘imminent dying parent'	4180
	#5	DE “parental death”	680
	#6	DE “death and dying” OR DE “parental death” OR DE “partner death”	35,038
	#7	#4 OR #5 OR #6	38,191
Outcomes Children’s strategies and (inter)action in their everyday life, and children’s and parents’ perspectives on these coping strategies and (inter)actions	#8	“Family life” OR “daily life” OR “family routine” OR “family functioning” OR “family function” OR “everyday life”OR “school life”OR “daily experience” OR “school time” OR “social time” OR “spare time” OR leisure OR socializing OR “social activity” OR “adverse childhood experience” OR “life change events “OR “life change event” OR “leisure activities” OR “adaptation psychological” OR “coping strateg*” OR “activities of daily living” OR “activity, daily living” OR ADL OR “daily living activity” OR hobbies OR hobby OR holidays OR “leisure activity” OR “leisure time” OR relaxation OR vacation OR “childhood adversity” OR “adverse childhood event” OR “adverse childhood experiences” OR “adverse experiences in childhood” OR “experiences in early childhood” OR “experiences in infancy” OR “child adverse experiences” OR “childhood adverse experience” OR “childhood adversities” OR “deleterious childhood experiences” OR “physiologic adaptation” OR “physiological adjustment” OR “coping behavior” OR “behavior coping” OR “coping ability” OR “coping behavior ” OR “coping mechanism” OR “coping strategy” OR “daily interaction” OR “family interaction” OR “interaction relationships” OR teachers OR teacher OR coach OR coaches OR “step parents” OR facilitator OR facilitators OR educator OR educators or “school teacher”	633,881
	#9	DE “family relations” OR DE “child discipline” OR DE “childrearing practices” OR DE “family conflict” OR DE “family separation” OR DE “intergenerational relations” OR DE “marital relations” OR DE “parent child relations” OR DE “parental role” OR DE “sibling relations” OR DE “transgenerational patterns” OR DE “family separation”	104,535
	#10	DE “family preservation” or DE “childhood adversity” OR DE “childhood development” OR DE “child behavior” OR DE “child characteristics” OR DE “child health” OR DE “early childhood development” OR DE “zone of proximal development”	100,774
	#11	DE “coping behavior” OR DE “coping style”	51,253
	#12	DE “social interaction” OR DE “conflict resolution” OR DE “encouragement” OR DE “interpersonal interaction” OR DE “interpersonal relationships” OR DE “nonviolence” OR DE “parasocial interaction” OR DE “peace” OR DE “personal space” OR DE “physical contact” OR DE “social exchange” OR DE “teamwork” OR DE “teasing” OR DE “victimization”	120,194
	#13	(DE “coaches”) OR (DE “teachers” OR DE “college teachers” OR DE “cooperating teachers” OR DE “elementary school teachers” OR DE “high school teachers” OR DE “junior high school teachers” OR DE “middle school teachers” OR DE “preschool teachers” OR DE “preservice teachers” OR DE “resource teachers” OR DE “special education teachers” OR DE “student teachers” OR DE “teacher characteristics” OR DE “vocational education teachers”)	91,663
	#14	DE “experiences (events)” OR DE “adversity” OR DE “anniversary events” OR DE “combat experience” OR DE “crises” OR DE “disasters” OR DE “early experience” OR DE “first experiences” OR DE “holidays” OR DE “life experiences” OR DE “life review” OR DE “traumatic experiences” OR DE “vicarious experiences”	83,771
	#15	#8 OR #9 OR #10 OR #11 OR #12 OR #13 OR #14	923,581
	#16	#3 AND #15	181,165
	#17	#7 AND 16	1859
	#18	#17 AND (limiters - publication year: 2012–2022)	539

Search from ERIC			
PEO		Search Terms (From Eric)	Search hits
Population Children <18 years old and children’s parents	#1	Juvenile OR juveniles OR minor OR minors OR adolescent OR adolescents OR teenager OR Child*OR “dependent child” OR youth OR “child preschool” OR “pre school child” OR siblings OR brother OR brothers OR sister OR sisters or children	168,646
	#2	DE “young children” OR DE “adolescents” OR DE “childhood needs” OR DE “elementary school students” OR DE “youth	135,636
	#3	DE “siblings” OR DE “twins”	3667
	#4	1 OR 2 OR 3	238,809
Exposure Live with a parent who is critically ill and dying	#5	“Deprivation parental “OR “deprivation paternal “OR “parent loss” OR “parent deprivation” OR “parental loss” OR “paternal deprivation” OR “dying parent” OR “dying parents” OR moribund OR “parent dying” OR “maternal deprivation” OR “imminent dying parent” OR “parental death” OR “paternal deprivation “OR “maternal deprivation” OR “parental deprivation” OR “prior to parent death” OR “attitude to parent death” OR “loss of parent*”or “death of parent*”or “parent bereavement” or death or dying or “paternal deprivation” or “maternal deprivation”	167
	#6	DE “terminal illness”	267
	#7	DE “death”	4826
	#8	#5 OR #6 OR #7	5048
Outcomes Children’s strategies and (inter)action in their everyday life, and children’s and parents’ perspectives on these coping strategies and (inter)actions	#9	“Family life” OR “daily life” OR “family routine” OR “family functioning” OR “family function” OR “everyday life”OR “school life”OR “daily experience” OR “school time” OR “social time” OR “spare time” OR leisure OR socializing OR “social activity” OR “adverse childhood experience” OR “life change events “OR “life change event” OR “leisure activities” OR “adaptation psychological” OR “coping strateg*” OR “activities of daily living” OR “activity, daily living” OR ADL OR “daily living activity” OR hobbies OR hobby OR holidays OR “leisure activity” OR “leisure time” OR relaxation OR vacation OR “childhood adversity” OR “adverse childhood event” OR “adverse childhood experiences” OR “adverse experiences in childhood” OR “experiences in early childhood” OR “experiences in infancy” OR “child adverse experiences” OR “childhood adverse experience” OR “childhood adversities” OR “deleterious childhood experiences” OR “physiologic adaptation” OR “physiological adjustment” OR “coping behavior” OR “behavior coping” OR “coping ability” OR “coping behavior ” OR “coping mechanism” OR “coping strategy” OR “daily interaction” OR “family interaction” OR “interaction relationships” OR teachers OR teacher OR coach OR coaches OR “step parents” OR facilitator OR facilitators OR educator OR educators or “school teacher”	670,961
	#10	DE “family life”	4525
	#11	DE “social experience”	687
	#12	DE “coping”	12,744
	#13	DE “relationship”	1769
	#14	DE “teacher educators”	8019
	#15	DE “family school relationship” OR DE “teacher participation”	9563
	#16	9 OR 10 OR 11 OR 12 OR 13 OR 14 OR 15	674,545
	#17	4 AND 8 AND 16	332
	#18	Limiters - date published: 20120101-20211231 no publi,cations ye,211,022	91

Search from MEDLINE			
PEO		Search Terms (From MEDLINE)	Search hits
Population Children <18 years old and children’s parents	#1	juvenile[Text word] OR juveniles[Text word] OR minor[Text word] OR minors[Text word] OR adolescent[Text word] OR adolescents[Text word] OR teenager[Text word] OR Child*[Text word] OR “dependent child”[Text word] OR Youth[Text word] OR “child Preschool”[Text word] OR “pre school child”[Text word] OR siblings[Text word] OR brother[Text word] OR brothers[Text word] OR sister[Text word] OR sisters[Text word] sort by: Most recent	4,091,846
	#2	“Child”[Mesh] OR “child, Preschool”[Mesh] OR “Adolescent”[Mesh] OR “Minors”[Mesh] OR “Child”[Mesh] OR “child, Preschool”[Mesh] OR “Adolescent”[Mesh] OR “Minors”[Mesh] OR “Siblings”[Mesh] sort by: Most recent	3,243,667
	#3	1 OR 2 (“Child”[Mesh] OR “child, Preschool”[Mesh] OR “Adolescent”[Mesh] OR “Minors”[Mesh] OR “Child”[Mesh] OR “child, Preschool”[Mesh] OR “Adolescent”[Mesh] OR “Minors”[Mesh] OR “Siblings”[Mesh]) OR (juvenile[Text word] OR juveniles[Text word] OR minor[Text word] OR minors[Text word] OR adolescent[Text word] OR adolescents[Text word] OR teenager[Text word] OR Child*[Text word] OR “dependent child”[Text word] OR Youth[Text word] OR “child Preschool”[Text word] OR “pre school child”[Text word] OR siblings[Text word] OR brother[Text word] OR brothers[Text word] OR sister[Text word] OR sisters[Text word]) sort by: Most recent	4,091,846
Exposure Live with a parent who is critically ill and dying	#4	“Deprivation parental”[Text word] OR “deprivation paternal”[Text word] OR “parent loss”[Text word] OR “parent deprivation”[Text word] OR “parental loss”[Text word] OR “paternal deprivation”[Text word] OR “dying parent”[Text word] OR “dying parents”[Text word] OR moribund[Text word] OR “parent dying”[Text word] OR “maternal deprivation”[Text word] OR “imminent dying parent”[Text word] OR “parental death”[Text word] OR “paternal deprivation”[Text word] OR “maternal deprivation”[Text word] OR “parental deprivation”[Text word] OR “prior to parent death”[Text word] OR “attitude to parent death”[Text word] OR “loss of parent*”or “death of parent*”or “parent bereavement”[Text word] or death[Text word] or dying[Text word] sort by: Most recent	921,693
	#5	“Death”[Mesh] or “paternal Deprivation”[Mesh] or “maternal Deprivation”[Mesh] sort by: Most recent	161,234
	#6	4 OR 5 (“Deprivation parental”[Text word] OR “deprivation paternal”[Text word] OR “parent loss”[Text word] OR “parent deprivation”[Text word] OR “parental loss”[Text word] OR “paternal deprivation”[Text word] OR “dying parent”[Text word] OR “dying parents”[Text word] OR moribund[Text word] OR “parent dying”[Text word] OR “maternal deprivation”[Text word] OR “imminent dying parent”[Text word] OR “parental death”[Text word] OR “paternal deprivation”[Text word] OR “maternal deprivation”[Text word] OR “parental deprivation”[Text word] OR “prior to parent death”[Text word] OR “attitude to parent death”[Text word] OR “loss of parent*”or “death of parent*”or “parent bereavement”[Text word] or (death[Text word] and (*N* = 5)parent*[Text word] or dying[Text word]) OR (“Death”[Mesh] or “paternal Deprivation”[Mesh] or “maternal Deprivation”[Mesh]) sort by: Most recent	981,304
Outcomes Children’s strategies and (inter)action in their everyday life, and children’s and parents’ perspectives on these coping strategies and (inter)actions	#7	“Family life”[Text word] OR “daily life”[Text word] OR “family routine”[Text word] OR “family functioning”[Text word] OR “family function”[Text word] OR “everyday life”[Text word] OR “school life”[Text word] OR “daily experience”[Text word] OR “school time”[Text word] OR “social time”[Text word] OR “spare time”[Text word] OR leisure[Text word] OR socializing[Text word] OR “social activity”[Text word] OR “adverse childhood experience”[Text word] OR “life change events”[Text word] OR “life change event”[Text word] OR “leisure activities”[Text word] OR “adaptation psychological”[Text word] OR “coping strateg*”[Text word] OR “activities of daily living”[Text word] OR “activity, daily living”[Text word] OR ADL[Text word] OR “daily living activity”[Text word] OR hobbies[Text word] OR hobby[Text word] OR holidays[Text word] OR “leisure activity”[Text word] OR “leisure time”[Text word] OR relaxation[Text word] OR vacation[Text word] OR “childhood adversity”[Text word] OR “adverse childhood event”[Text word] OR “adverse childhood experiences”[Text word] OR “adverse experiences in childhood”[Text word] OR “experiences in early childhood”[Text word] OR “experiences in infancy”[Text word] OR “child adverse experiences”[Text word] OR “childhood adverse experience”[Text word] OR “childhood adversities”[Text word] OR “deleterious childhood experiences”[Text word] OR “physiologic adaptation”[Text word] OR “physiological adjustment”[Text word] OR “coping behavior”[Text word] OR “behavior coping”[Text word] OR “coping ability”[Text word] OR “coping behavior ”[text word] OR “coping mechanism”[Text word] OR “coping strategy”[Text word] OR “daily interaction”[Text word] OR “family interaction”[Text word] OR “interaction relationships”[Text word] OR teachers[Text word] OR teacher[Text word] OR coach[Text word] OR coaches[Text word] OR “step parents”[Text word] OR facilitator[Text word] OR facilitators[Text word] OR educator[Text word] OR educators[Text word] sort by: Most recent	2,453,903
	#8	“Adverse childhood Experiences”[Mesh] or “adaptation, psychological”[Mesh] or “school Teachers”[Mesh] sort by: Most recent	139,611
	#9	7 OR 8 (“Family life”[Text word] OR “daily life”[Text word] OR “family routine”[Text word] OR “family functioning”[Text word] OR “family function”[Text word] OR “everyday life”[Text word] OR “school life”[Text word] OR “daily experience”[Text word] OR “school time”[Text word] OR “social time”[Text word] OR “spare time”[Text word] OR leisure[Text word] OR socializing[Text word] OR “social activity”[Text word] OR “adverse childhood experience”[Text word] OR “life change events”[Text word] OR “life change event”[Text word] OR “leisure activities”[Text word] OR “adaptation psychological”[Text word] OR “coping strateg*”[Text word] OR “activities of daily living”[Text word] OR “activity, daily living”[Text word] OR ADL[Text word] OR “daily living activity”[Text word] OR hobbies[Text word] OR hobby[Text word] OR holidays[Text word] OR “leisure activity”[Text word] OR “leisure time”[Text word] OR relaxation[Text word] OR vacation[Text word] OR “childhood adversity”[Text word] OR “adverse childhood event”[Text word] OR “adverse childhood experiences”[Text word] OR “adverse experiences in childhood”[Text word] OR “experiences in early childhood”[Text word] OR “experiences in infancy”[Text word] OR “child adverse experiences”[Text word] OR “childhood adverse experience”[Text word] OR “childhood adversities”[Text word] OR “deleterious childhood experiences”[Text word] OR “physiologic adaptation”[Text word] OR “physiological adjustment”[Text word] OR “coping behavior”[Text word] OR “behavior coping”[Text word] OR “coping ability”[Text word] OR “coping behavior ”[text word] OR “coping mechanism”[Text word] OR “coping strategy”[Text word] OR “daily interaction”[Text word] OR “family interaction”[Text word] OR “interaction relationships”[Text word] OR teachers[Text word] OR teacher[Text word] OR coach[Text word] OR coaches[Text word] OR “step parents”[Text word] OR facilitator[Text word] OR facilitators[Text word] OR educator[Text word] OR educators[Text word]) OR (“adverse childhood Experiences”[Mesh] or “adaptation, psychological”[Mesh] or “school Teachers”[Mesh]) sort by: Most recent	562,890
#10	3 AND 6 AND 9 (((“Family life”[Text word] OR “daily life”[Text word] OR “family routine”[Text word] OR “family functioning”[Text word] OR “family function”[Text word] OR “everyday life”[Text word] OR “school life”[Text word] OR “daily experience”[Text word] OR “school time”[Text word] OR “social time”[Text word] OR “spare time”[Text word] OR leisure[Text word] OR socializing[Text word] OR “social activity”[Text word] OR “adverse childhood experience”[Text word] OR “life change events”[Text word] OR “life change event”[Text word] OR “leisure activities”[Text word] OR “adaptation psychological”[Text word] OR “coping strateg*”[Text word] OR “activities of daily living”[Text word] OR “activity, daily living”[Text word] OR ADL[Text word] OR “daily living activity”[Text word] OR hobbies[Text word] OR hobby[Text word] OR holidays[Text word] OR “leisure activity”[Text word] OR “leisure time”[Text word] OR relaxation[Text word] OR vacation[Text word] OR “childhood adversity”[Text word] OR “adverse childhood event”[Text word] OR “adverse childhood experiences”[Text word] OR “adverse experiences in childhood”[Text word] OR “experiences in early childhood”[Text word] OR “experiences in infancy”[Text word] OR “child adverse experiences”[Text word] OR “childhood adverse experience”[Text word] OR “childhood adversities”[Text word] OR “deleterious childhood experiences”[Text word] OR “physiologic adaptation”[Text word] OR “physiological adjustment”[Text word] OR “coping behavior”[Text word] OR “behavior coping”[Text word] OR “coping ability”[Text word] OR “coping behavior ”[text word] OR “coping mechanism”[Text word] OR “coping strategy”[Text word] OR “daily interaction”[Text word] OR “family interaction”[Text word] OR “interaction relationships”[Text word] OR teachers[Text word] OR teacher[Text word] OR coach[Text word] OR coaches[Text word] OR “step parents”[Text word] OR facilitator[Text word] OR facilitators[Text word] OR educator[Text word] OR educators[Text word]) OR (“adverse childhood Experiences”[Mesh] or “adaptation, psychological”[Mesh] or “school Teachers”[Mesh])) AND ((“Child”[Mesh] OR “child, Preschool”[Mesh] OR “Adolescent”[Mesh] OR “Minors”[Mesh] OR “Child”[Mesh] OR “child, Preschool”[Mesh] OR “Adolescent”[Mesh] OR “Minors”[Mesh] OR “Siblings”[Mesh]) OR (juvenile[Text word] OR juveniles[Text word] OR minor[Text word] OR minors[Text word] OR adolescent[Text word] OR adolescents[Text word] OR teenager[Text word] OR Child*[Text word] OR “dependent child”[Text word] OR Youth[Text word] OR “child Preschool”[Text word] OR “pre school child”[Text word] OR siblings[Text word] OR brother[Text word] OR brothers[Text word] OR sister[Text word] OR sisters[Text word]))) AND ((“deprivation parental”[Text word] OR “deprivation paternal”[Text word] OR “parent loss”[Text word] OR “parent deprivation”[Text word] OR “parental loss”[Text word] OR “paternal deprivation”[Text word] OR “dying parent”[Text word] OR “dying parents”[Text word] OR moribund[Text word] OR “parent dying”[Text word] OR “maternal deprivation”[Text word] OR “imminent dying parent”[Text word] OR “parental death”[Text word] OR “paternal deprivation”[Text word] OR “maternal deprivation”[Text word] OR “parental deprivation”[Text word] OR “prior to parent death”[Text word] OR “attitude to parent death”[Text word] OR “loss of parent*”or “death of parent*”or “parent bereavement”[Text word] or death[Text word] or dying[Text word]) OR (“Death”[Mesh] or “paternal Deprivation”[Mesh] or “maternal Deprivation”[Mesh])) sort by: Most recent	4236
#11	#10 AND **filters: From 2012 - 2022** sort by: Most recent	1730

### Inclusion and Exclusion Criteria

The inclusion criteria were: Empirical qualitative research studies; studies including populations of children <18 years old or/and their parents; studies reporting on children’s strategies and (interactions) in everyday life or/and parents’ perspectives on children’s strategies in everyday life while having a critically ill parent. Critically ill is defined as a medical condition that is advanced, progressive, incurable and life-limiting. The search was limited to empirical qualitative research studies written in English and published from 1 January 2012 to 31 December 2021. The following studies were excluded: Literature reviews, editorials, abstract only, case studies, quantitative design studies, published in languages other than English.

### Selecting, Appraisal and Extracting Relevant Data

**Figure 1.**
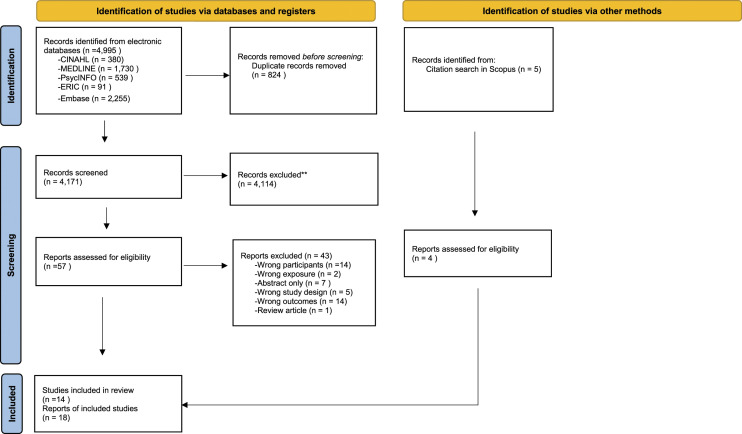
PRISMA 2020 flow diagram for new systematic reviews which included searches of databases, registers and other sources. *Consider, if feasible to do so, reporting the number of records identified from each database or register searched (rather than the total number across all databases/registers). **If automation tools were used, indicate how many records were excluded by a human and how many were excluded by automation tools. From: Page MJ, McKenzie JE, Bossuyt PM, Boutron I, Hoffmann TC, Mulrow CD, et al. The PRISMA 2020 statement: an updated guideline for reporting systematic reviews. BMJ 2021; 372:*n*71. doi: 10.1136/bmj.*n*71. For more information, visit: http://www.prisma-statement.org/

The initial search from five electronic databases identified 4995 studies, which were transferred to Covidence software for the following screening process (Veritas Health Innovation, 2020). After the removal of duplicates and titles and abstracts screening, 57 studies were eligible for full text review. Of these, 14 studies met the inclusion criteria. Additionally, four studies were identified through pearl growing citation in Scopus, resulting in a total of 18 studies being included in the current review. The entire study selection process was conducted collaboratively by two of the authors (CF and HX). In case of disagreement in the screening or full-text review process, the two authors (CF and HX) discussed with the two other authors (SG and SS) until an agreement was reached. All four authors agreed on the included studies. The selection process was carried out in accordance with the PRISMA guidelines and reasons for exclusion of studies were recorded (Page et al., 2021). The study selection process was presented in a PRISMA flow diagram (Figure 1).

The Critical Appraisal Skills Program (CASP) qualitative study checklist was used to appraise the quality of included studies (CASP UK, 2018). The checklist consisted of ten questions assessing the studies’ aim, methodology and design, recruitment strategy, data collection, data analysis, findings, and research value, see Table 3. No studies were excluded in this process.

**Table 3.** CASP Quality assessment of included studies.

Authors	Question Items from Critical Appraisal Skills Program Checklist									
	1. Was there a Clear Statement of the Aims of the Research?	2. Is a Qualitative Methodology Appropriate?	3. Was the Research Design Appropriate to Address the Aims of the Research?	4. Was the Recruitment Strategy Appropriate to the Aims of the Research?	5. Was the Data Collected in a way that Addressed the Research Issue?	6. Has the Relationship Between Researcher and Participant been Adequately Considered?	7. Have Ethical Issues been Taken into Consideration?	8. Was the Data Analyses Sufficiently Rigorous?	9. Is there a Clear Statement of Findings?	10. How Valuable is the Research?
Buchwald et al. (2012)	yes	yes	yes	yes	yes	yes	yes	yes	yes	yes
Fearnley (2012)	yes	yes	yes	yes	yes	no, not discussed researcher’s role or potential bias	yes	no, limited description in the analysis process	no, no discussion of credibility and the evidence both for and against the authors’ arguments	no, not discussed implications or transferability
Fearnely and Boland (2019)	yes	yes	yes	yes	yes	yes	yes	yes	yes	yes
Graungaard et al. (2021)	yes	yes	yes	yes	yes	no	yes	yes	yes	yes
Keeley and Baldwin (2012)	yes	yes	yes	yes	yes	no, not mentioned researcher’s bias	yes	yes	yes	Yes
Keeley and Generous (2013)	yes	yes	yes	yes	yes	no, not discussed researcher’s own role or potential bias	yes	yes	yes	Yes
Lytje et al. (2022)	yes	yes	yes	yes	yes	yes	yes	yes	yes	yes
Lytje Dyregrov (2021)	yes	yes	yes	yes	yes	no, did not discuss researcher’s role or bias	yes	yes	yes	yes
Marcusson et al. (2020)	yes	yes	yes	yes	yes	yes, reflected research’s role and	yes	yes	yes	yes
Mayo et al. (2016)	yes	yes	yes	yes	yes	no, limited details	yes	yes	yes	yes
Melcher et al. (2015)	yes	yes	yes	yes	yes	yes	yes	yes	yes	yes
Sheehan et al. (2014)	yes	yes	yes	yes	yes	no, reflected research design but not discussed researcher’s own bias	yes	yes	yes	yes
Sheehan et al. (2016)	yes	yes	yes	yes	yes	no	yes	yes	yes	yes
Stephenson et al. (2015)	yes	yes	yes	yes	yes	no, not discussed researcher’s role or potential bias	yes	yes	yes	yes
Sven et al. (2015)	yes	yes	yes	yes	yes	yes, reflected research’s role on sample recruitment and choice of location	yes	yes	yes	yes
Turner (2017)	yes	yes	yes	yes	yes	yes	yes	yes	yes	yes
Turner (2020)	yes	yes	yes	yes	yes	no	yes	yes	yes	yes
Turner and Almack (2017)	yes	yes	yes	yes	yes	yes, reflected researcher’s role in data collection	yes	yes	yes	yes

The 18 included studies were marked with an asterisk * in the references. The following information was extracted from the articles: 1) Authors, 2) Location, 3) Journal, 4) Impact factor (extracted from journal website), 5) Study period, 6) Study design, 7) Sample size, 8) Target group and context, 9) Theory/concepts, 10) Results, and 11) Limitations. A selection of the data is presented in Table 4. The extracted data was checked for accuracy by all four authors.

**Table 4.** Study Characteristics.

Author(s)Study LocationYear of Publication	Journal (Year: Impact Factor)	Aim(s) of the Study	Study Populations’ Characteristics	Method(s) Theory/Concept (Time Period)	Main Findings	Limitations
Buchwald et al. Sweden 2012	Scandinavian journal of caring sciences (2012:IF 2.340)	To describe and understand how children handle their life when a mother or father is dying	Seven children (age 11-17, avg known) Parental illness: N/A	Qualitative interviews (length unknown) and video diaries Analysis based on phenomenological theory (Time period N/A)	Analysis produced an overarching theme of death’s waiting room, with following subthemes: Relating to death, when death becomes even more clearly manifest and handling life in death’s waiting room. The findings show that children fear death most of all when a parent becomes seriously ill even though they are not told that the parent is dying. Children had many thoughts about death and losing a parent and used various ways of handling them. Parents who did not discuss death with children, leading them to feel alone and unprepared for the imminent loss of a parent	The study was limited to focusing on children’s life world. It did not include the relational aspects that were essential aspects of children’s lives Thel ethnic profile of participants were not required, and the sample of participants were small, which may limit the transferability to other contexts
Fearnely The United Kingdom 2012	Journal of community practice (2012: IF 1.063)	To explore children’s experiences when living with a parent who is dying	Seven children and young people (age9-24 at the time of interview, but lived with their ill parent <18, avg unknown) participated in interview Eight children (age 7-15, avg known) participated in focus group Sex known Parental illness: N/A	Semi-structured interview (length unknown) and a focus group (length unknown) Template analysis (Time period N/A)	Children wanted to be included in conversations when parents were at the end of life. The quality and quantity of information given to children corresponded with how well they coped with everyday life during the parents’ illness and after the loss of parents. At times, children actively sought information about ill parent’s disease and imminent death, but their attempts were obstacles, possibly due to parents’ and professionals’ reluctance to integrate them	Limited information about the study’s participant’s characteristics
Fearnely & Boland The United Kingdom 2019	Healthcare (2019: IF 2.645)	To explore children’s experiences of living with a parent who had life-limiting Illness, and professionals’ perspectives of the support offered to children	Seven children (age 9–24, avg 14, at the time of interview, but lost their parents <18), and 16 professionals Five females Two male Parental illness: Cancer MultipleSclerosis, neurone disease	Semi-structured interview (40–120 min, avg unknown) Thematic analysis (Time period N/A)	The complex inter-relationship of communication between parents, who have a life-limiting illness, their children, and professionals involved in their care has been explored. From the children’s perspectives, it was evident that they all knew that their parents were ill but did not always know the extent of the illness and prognosis. The quality and quantity of information about the illness was important for how they remembered the illness period and importantly how they managed their distress during this time	Retrospective interview The age range of children and the length of time since bereavement varied
Graungaard et al. Denmark 2021	Journal of psychosocial oncology 2021: IF 2.029	To explore the difficulties parents face when understanding their children’s reactions to parental cancer and parents’ reactions to their children’s perceived needs	Eleven parents with cancer and seven partners Age: 30-50 (avg unknown) (All their children, age1–15) Eight females, one male Parental illness: Cancer	Semi-structured interviews (90–180 min, avg unknown) and field notes Inductive analysis with systematic Text condensation (Time period N/A)	Parents were groping in the dark when evaluating the wellbeing of their children. Parents observed physical, mental and social signs of difficulty in their children but were in doubt on how to react. The most common way to cope with the uncertainty and fear of the effect of the disease on the children’s lives was to minimise changes in daily life and to normalise the behaviour of the children. Parents applied individually based strategies for communicating with their children. All parents described a severe lack of acceptable and relevant formal support offers for the family as a unit	The sample was primarily an urban, ethnically western population with secondary or higher education, and most of the patients were women and living in couples. These characteristics decreased the transferability of the findings This study presented cross-sectional data that limited the possibility to evaluate parents’ experiences over time
Keeley & Baldwin The United States of America 2012	Journal of loss and trauma (2012: 1.063)	To examine messages of everyday communication and their importance in the family at the end of life	Sixty-one children (age 6–18, avg 13) Forty-one females and 20 males Parental illness: N/A	Semi-structured interviews (5–42 min, avg 10min) Inductive approach analysis based on grounded theory Social construction theory (Time period N/A)	Everyday communication conducted during daily and routine interactions, provided a shield of safety and comfort for children. Spending time together with their dying parents was children’s way to demonstrate the significance of the family relationship for both the children and the dying parent. Some daily routines became family rituals, which functioned as a buffer, balancing stress and transition to the new situation	Seventy-two percentage of the sample were caucasian The interviews were short
Keely & Generous The United States of America 2014	Death studies (2014: IF 2.245)	To understand more about final conversations (communication between loved ones from the point of terminal diagnosis until death)	Forty-nine children and adolescents (age 7-18, avg 12) who had final conservation with dying loved ones Thirty-one females, 18 males Parental illness: N/A	Semi-structured interview (5–42 min, avg 14 min) Inductive analysis (Time period N/A)	Children were altruistic and spent as much time as possible with their dying parent, supporting and communicating with the parent, including expressions of love as a way to maintain a sense of normality in everyday life Children needed open and honest communication regarding their ill parent’s impending death. The ill parent’s personal histories and stories were significant for some children at present and in the future Some teenagers sought help from external support networks to talk about the parent’s impending death and related concerns, which served as an emotional shelter for them	Lack of gender or cultural diversity. Most participants were white girls
Lytje & Dyregrov Denmark 2021	Journal of death and dying (2021: IF 1.953)	To explore how parents understand the needs and grief of their children and how to support children when family experiences critical Parental illness and death	Thirteen remaining parents who had dependent children (age 3-6, avg age unknown, when the co-parent died) Sex unknown Parental illness: Cancer and neurodegenerative disorder	Semi-structured interviews (40–60 min, avg unknown) Thematic analysis (Time period N/A)	From the perspective of parents, it was difficult to inform children about a parent’s illness and impending death, as parents often were unsure about the future. Some parents believed it was not possible to hide the situation from children, as they found children sought for information independently, e.g., by overhearing the conversation between parents and other relatives. Other parents who did not tell their children about the ill parent’s situation, found their child was unhappy about it. Parents witnessed a range of grief reactions and coping mechanisms related to their children	Small sample consisted exclusively of couples who were together at the time of the loss, which might not reflect the experience in single-parent households
Lytje et al. Denmark 2022	International journal of early years education (2022: IF 1.54)	To investigate young children’s experiences living in a family with critical ill parent and following the parent’s death	Twelve children (age 5-8, avg unknown) At the time of the interview Sex unknown Parental illness: Cancer, neurodegenerative disorder	Children tailored sand-tray interviews with interview guide (30–50 min, avg unknown) Thematic analysis (Time period N/A)	Children wanted truthful information about their parent’s illness. They wanted to be included in decisions and rituals, and they wanted to understand their loss. They appreciated support from and conversations with the remaining parent, the daycare staff, and friends. Young children must be supported in understanding the new situation in their lives	The young age of participants might have limited their ability to express what they experienced The convenience sample might exclude the experience of other children who had no energy to participate in it
Marcusson et al. Denmark 2020	Journal of clinical nursing (2019: IF 3.036)	To explore how children and young adults from divorced families experience double bereavement when they lose a divorced parent with cancer and how the double bereavement influences their mental health consequences and need of support	Fifty-eight participants, here of 18 children (age 7-15, avg unknown) Sex unknown Parental illness: Cancer	Three hundred forty hours participant observations and individual interviews (25–60 min, avg unknown) Analysis was inspired by Ricoeur’s phenomenological Hermeneutical interpretation theory Stroebe and Schut’s coping model, “the dual process modelof coping with bereavement” (Jan 2017 - Jun 2018)	This study highlights dimensions of double bereavement result- Ing from divorced parental cancer and death. Double bereavement can also be described as the disruptions within, and between, the two family worlds including a lack of information about illness and deterioration-related changes to life routines and close relationships. Young children were forced to navigate and to cope with disruptions between two family worlds.. They adopted either an active caregiving role or a passive observer role. While some children found meaning from caregiving experience, other children who adopted an observant role, avoided talking about their parent’s illness, experienced higher levels of distress. Accessible support in and between two family worlds can be beneficial for double bereaved children	Study was conducted in one cancer setting, and mainly focused on children from a divorced family followed by a parent death, which might only represent certain socio-demographic groups The participants had skewed gender distribution The researcher developed a relationship over time with the participants, which could have biassed the results
Mayo et al. The United States of America 2016	Journal of hospice & palliative nursing (2016: IF 1.918)	To identify the type of interactions the adolescents had with members of a hospice healthcare team	Thirty adolescents whose parents received hospice services at local facilities (age 12-18, avg 15) 18 females, 12 males Parental illness: Mostly cancer diagnosis	Individual interviews (avg 45 min) and field notes A second interview after the death of the ill parent was conducted with six families (length unknown) Content analysis (Dec 2010 - Jul 2012)	Four types of interactions between teenagers living with a dying parent and palliative healthcare professionals were identified: No interactions, in-passing interactions, engaged interactions, and formal interactions. Most of the teenagers had no interaction or very limited interaction with healthcare professionals. Only a few teenagers engaged in interactions with healthcare professionals, or received formal support from them. The limited contact occurred due to logistics or that healthcare professionals excluded children in the patient care	The sample may only represent teenagers who were willing to talk about parent’s illness and imminent death
Melcher et al. Sweden 2015	Palliative & supportive care (2015: IF 2.257)	To describe how teenagers are emotionally affected by everyday life in a family with a dying parent and to determine how they attempt to adapt to this situation	Ten teenagers (age 14–19 years, avg unknown) Three females, seven males Parental illness: Mostly cancer diagnosis	Two individual informal interview (I1 30–50 min (avg unknown); I2: 10–40 minutes(avg unknown) Content analysis (Mar - May 2013)	Teenagers tried to understand their parent’s illness by interpreting the information they received or observing their parent’s physical appearances. They strived to adjust strategies in order to support their ill parent. Teenagers took on domestic responsibility for supporting their parents and siblings, and maintaining family life such as cleaning and caring for pets. Parents’ avoidance of talking about illness and death resulted in teenagers having to guess and to interpret the vague signs of parent’s failing health on their own, and left them feeling lonely and unprepared. The school environment was of great importance for the teenagers to maintain their ‘usual’ everyday life	Interviews were made retrospectively There is a large variation in terms of the length of time after a parent’s death that interviews took place
Sheehan et al. The United States of America 2014	Journal of palliative medicine (2014: IF 2.947)	To explore the complex and dynamic ways in which disclosure occurs when a parent is in Hospice	Fifty-six family members from a hospice program (20 not-ill parents, 10 ill parents, 26 adolescents) Adolescents' age: 12-18, avg 15 Not-ill parents’ age: 34-60, avg 50 Ill parents’ age: 40-62, avg 55 Adolescents: 16 females, 10 males Parents: 20 females,10 males Parental illness: Mostly cancer diagnosis	Individual interviews (length unknown) and field notes In-depth analysis (Dec 2010 - Jul 2012)	Families with a dying parent engaged their children in the process of disclosure in one of four ways: measured telling, skirted telling, matter-of-fact telling, and inconsistent telling. In families with good dynamics, measured telling and skirted telling were often used, where teenagers considered what information they wanted or needed, proactively letting their parents know what they were ready to hear or what they did not want to discuss. In families with complicated relationships, some teenagers applied a passive way to receive the information and reveal to parents they understand the realities of the illness, while other teenagers accepted the parent’s critical illness without acknowledging the parent was dying	Only one hospice facility. Family characteristics were not explored
Sheehan et al. Northeastern Ohio, the United States of America 2016	Palliative & supportive care (2016:IF 2.257)	To generate an explanatory model of the coping strategies that adolescents employ to manage the stressors they experience in the final months of their ill parent’s life and shortly after their death	Sixty-one participants from 26 families from a hospice program (30 adolescents, 11 ill parents and 20 not-ill parents) Adolescents' age: 12-18 (avg 15) Not-ill parent’s age: 34-60 (avg 50) Ill parents’ age: 40-62 (avg 55) Adolescents: 16 female 10 males Parents: 20 female 10 males Parental illness: Mostly cancer diagnosis	Semi-structured interviews before (length unknown), and after the parent’s death (length unknown) Analysis inspired by grounded theory (Dec 2010 - Jul 2012)	When a parent was dying, the overarching challenge for teenagers was the process of managing and integrating the ‘two worlds’- the well world and the ill world. The teenagers had strategies of 1) maintaining normality as usual, 2) using the distraction of normal activities to avoid worry, 3) providing care for the ill parent and younger siblings, 4) taking on household responsibilities, and 5) talking about their parent’s illness with their friends or siblings	Sample was drawn from a single hospice facility The sample size did not explore variation in family characteristics
Stephenson et al. The United States of America F2015	International journal of palliative nursing (2015: IF 0.718)	To examine uncertainty as a salient theme for families in which a parent was dying while receiving hospice care	Sixty-one participants from 26 families receiving hospice service (30 adolescents, 11 ill parents, and 20 not-ill parents) Adolescents' age: 12-18 (avg 15) Not-ill parent’s age: 34-60 (avg 50) Ill parents’ age: 40-62 (avg 55) Adolescents: 16 female 10 males Parents: 20 female 10 males Parental illness: Cancer	Interviews before (length unknown) and after parent’s death (length unknown) Content analysis (Dec 2010 - Jul 2012)	All participants expressed uncertainty. Some children found uncertainty about parent’s illness and imminent death due to their parents being reluctant or having difficulties in communicating with the children. Some teenagers managed the uncertainty through their own information seeking, such as reading, searching the internet, or asking information from co-parents in relation to their ill parent’s situation. However, the strategy of getting information from co-parents was sometimes difficult. Other teenagers used avoidance as a strategy to manage the uncertainty as a kind of self-protection against the harsh reality of losing a parent	Included primarily christian families living in midwestern US, which may limit the study’s transferability to other contexts
Sven et al. Sweden 2016	Palliative and supportive care (2016: IF 1.968)	To explore how teenagers reason about a parent’s recent death and about their life without that parent	Ten teenagers (age 14–19, avg known) Three females, seven males Parental illness: Cancer	Two times of individual, informal interviews (The first interview 30–50 min (avg unknown); the second interview 10–40 min, avg unknown)Content analysis (Mar - May 2013)	During a parent’s critical illness course, some teenagers choose to stay with their ill parent as a support, but others found it was difficult to witness parent’s deteoritating, although they wanted to be present and included. Teenagers managed their situations in different ways: some talked with friends, school counsellors, or co-parent during the parent’s illness and after the loss of a parent, while others chose not talking about it as a strategy to keep life as usual	Retrospective interview The sample of teenagers might only represent a small group of children who coped well
Turner The United Kingdom 2018	Palliative and supportive care (2018: IF 1.968)	To explore young people’s experiences of family interaction when a parent is dying	Ten young people living (age 13-21, avg 17) with a parent, expected death within next 12 months Sex unknown Parental illness: Cancer 9 Neurone disease 1	Individual semi-structured interview (length unknown) Voice-centred relational analysis method (Time period N/A)	Young people’s attitudes toward open communication between family members were ambivalent and ambiguous. Some young people valued open and honest communication, as this reinforced the close relationships between family members, while others wanted to exercise more control over their knowledge of parental illness and prognosis. Having a choice about how much information they receive may facilitate the ability to move in and out of a state of open awareness and enable young people to better accommodate the experience of parental illness in their everyday lives	Small sample size
Turner The United Kingdom 2020	Sociology of health & illness (2020: IF3.041)	To explore how young people who are living with a parent who is dying talk about the future	Ten young people (age 13-21, avg unknown) Three females, seven males Parental illness: N/A	Individual interview (25–80 min, avg unknown) Gilligan’s voice-centred relational analysis Data collected periodN/A Two conceptual model: Biographical disruption and continuing bonds (Time period N/A)	Young people were able to move imaginatively beyond the imminent death of a parent, maintaining a sense of biographical continuity. Using the strategy of thinking about the future, most young people were able to generate an alternative to the ‘harm story’ typically associated with parental loss. By integrating the parent’s illness and impending death in the present, young people were able to make sense of living with the dying, and furthermore, it gave meaning to their imagined futures	Small sample size of participants. The study was carried out retrospectively because of the challenge of involving young people when a parent is dying
Turner & Almack The United Kingdom 2019	Children’s geographies (2019: IF 2.291)	To explore young people’s experiences of living with a Parent who is at the end of life and how family lives are troubled by life-limiting Parental illness	Ten young people (age 13-21, avg 17) Three females, seven males Parental illness: Cancer and neurone disease	Individual, semi-structured interviews (length unknown) Data were analysed using voice-centred relational methods (Time period N/A)	The young people’s everyday life of living with a dying parent was impacted by the presence/absence of the significant ones. They recognised the changes in family dynamics due to a parent’s critical illness, and some of them responded by taking on more responsibility to provide care for family members and for themselves	The study took young people’s experience alone and did not provide relational perspectives on young people’s everyday lives

### Strategy for Data Analysis

The analytical synthesis of data consisted of a descriptive summary analysis presented as ‘*Characteristics of the studies’* and a thematic analysis inspired by Braun and Clarke (2006). Firstly, the authors read and re-read the included studies to familiarise with the material. The key findings from the included studies were summarised descriptively in a working table. Secondly, authors independently identified the phrases and excerpts, also called data items, from the included studies, which described children’s strategies and interaction in their everyday life. These data items were further condensed into codes. Thirdly, the coded material was categorised into groups based on the commonalities of the coded material, and initial sub-themes were constructed. Finally, the two themes were further developed in a consensual process of analysis amongst the authors in order to present a pattern of data from the included studies. A process of data checking was conducted between the constructed themes, in order to ensure all themes adequately reflected the empirical material. Thus, the two final themes with two and three subthemes, respectively, were refined, defined, and named, see Table 5.

**Table 5.** Themes and sub-themes.

Themes	Sub-themes
Strategies for maintaining usual everyday living in an unusual life situation	Supporting and socialising with healthy and ill parents
	The children’s inner will and the parents’ initiatives for family communication
Different ways of constructing meaning in the face of a forthcoming loss	The expectation of family cohesiveness during difficult times
	Meaning-making of a parent’s illness and death
	The need of strangers’ support

## Results

### Characteristics of the Studies

The 18 included studies were published between 2012 and 2021, with seven studies specifying that data were collected from 2012 to 2020. However, 11 studies did not report data collection periods (Buchwald et al., 2012; Fearnely, 2012; Fearnley & Boland, 2019; Graungaard et al., 2021; Keeley & Baldwin, 2012; Keeley & Generous, 2013; Lytje & Dyregrov, 2021; Lytje et al., 2022; Turner, 2017; 2020; Turner & Almack, 2017). The included studies originated from the United States of America *(6)* (Keeley & Baldwin, 2012; Keeley & Generous, 2013; Mayo, 2016; Sheehan et al., 2014; 2016; Stephenson et al., 2015), the United Kingdom *(5)* (Fearnley, 2012; Fearnley & Boland, 2019; Turner, 2017; 2020; Turner & Almack, 2017), Sweden *(3)* (Buchwald et al., 2012; Melcher et al., 2015; Sveen et al., 2015), and Denmark *(4)* (Graungaard et al., 2021; Lytje et al., 2022; Lytje & Dyregrov, 2021; Marcussen et al., 2020). The studies were published in journals of palliative care and death studies *(7)*, general nursing and care studies *(3)*, palliative nursing *(2)*, loss and trauma *(1)*, sociology *(1)*, psycho-oncology *(1)*, community care *(1)*, and children and education *(2)*, respectively. The journals’ impact factor ranged from 0.718-3.036. Selected details of the included studies were summarised in Table 4.

Ten studies described children’s or young people’s (5–18 years) strategies and interactions while living with a critically ill parent in everyday life (Buchwald et al., 2012; Fearnley, 2012; Lytje et al., 2022; Marcussen et al., 2020; Melcher et al., 2015; Sheehan et al., 2016; Sveen et al., 2015; Turner, 2017; 2020; Turner & Almack, 2017). One study explored children’s worries and difficulties in family interactions from the ill parent’s perspective (Graungaard et al., 2021), and one study described children’s everyday needs and support from the surviving parent’s perspective (Lytje & Dyregrov, 2021). Two studies explored the children’s everyday interactions by focusing on the family’s end-of-life communication from the children’s perspectives (Keeley & Baldwin, 2012; Keeley & Generous, 2013), one study focused on strategies and interactions around disclosing parents’ disease to children from both parents’ and children’s perspectives (Sheehan et al., 2014), and one study explored how family members including children cope with uncertainties when a parent is dying (Stephenson et al., 2015). Further, two studies described the children’s interaction with hospice providers in their everyday lives while having a critically ill parent from the children’s perspectives (Fearnley & Boland, 2019; Mayo, 2016). All studies concerned children’s everyday lives. Altogether, the studies included 123 parents (both healthy and ill parents), and 318 children (age 5-24 at the time of the studies). However, all children had lost a parent or lived with a critically ill and dying parent before the age of 18. In 16 studies, cancer was the most prevalent parental disease, along with neurodegenerative diseases. Two studies did not report parental disease (Keeley & Baldwin, 2012; Keeley & Generous, 2013). All studies had qualitative research designs, whereof nine studies used semi-structured interviews (Fearnley & Boland, 2019; Keeley & Baldwin, 2012; Keeley & Generous, 2013; Lytje & Dyregrov, 2021; Sheehan et al., 2016; Stephenson et al., 2015; Turner, 2017; 2020; Turner & Almack, 2017), four studies combined field observations and individual interviews (Graungaard et al., 2021; Marcussen et al., 2020; Mayo, 2016; Sheehan et al., 2014), two studies were conducted using informal interviews as free-ranging conversations (Melcher et al., 2015; Sveen et al., 2015), one study integrated individual interviews and video diaries (Buchwald et al., 2012), one study used semi-structured interviews and focus group interviews (Fearnley, 2012), and one study used sand-tray interview techniques, which was designed especially for young children (Lytje et al., 2022). Four studies worked within theoretical frameworks or conceptual models to guide their research questions and data analysis (Buchwald et al., 2012; Keeley & Baldwin, 2012; Marcussen et al., 2020; Turner, 2020). All studies described the interview questions, whereof three studies provided a full interview guide (Graungaard et al., 2021; Keeley & Baldwin, 2012; Lytje et al., 2022). All studies considered international ethical standards. Furthermore, all studies had clearly analytic strategies, with only one study lacking clarity in both analysis strategy and study findings (Fearnley, 2012). A varied number of limitations relating to the samples’ characteristics, such as ethnicity, family household situation, sample size, and transferability were reported in all studies despite one study not stating any limitations (Fearnley, 2012). Moreover, eight studies examined the researchers’ potential influence on the study (Buchwald et al., 2012; Fearnley & Boland, 2019; Lytje et al., 2022; Marcussen et al., 2020; Melcher et al., 2015; Sveen et al., 2015; Turner, 2017; Turner & Almack, 2017). The overall quality appraisal details of all included studies were presented in Table 3.

***Strategies for maintaining usual everyday living in an unusual life situation***

#### Supporting and Socialising with Healthy and ill Parents

From the children’s perspective, three studies showed that several children spent more time socialising with their parents while the parent was seriously ill (Buchwald, et al., 2012; Keeley & Generous, 2013; Sheehan et al., 2016) with a sharpened attention towards the family’s routine interactions such as sharing meals, playing games, reading bedtime stories together, participating in bath-time rituals, and completing household and outdoor activities together (Keeley & Generous, 2013). Children tried to support a positive and altruistic environment using verbal and non-verbal approaches, such as talking with parents, hugging the ill parent every day, as they believed this led to a good interaction with parents that were facing an impending death (Keeley & Generous, 2013). In Keeley and Baldwin’s (2012) study, children often had small-talks with dying parents on topics such as school, sports, or mutual hobbies. These daily conversations extended beyond creating a connection between children and dying parents into a family ritual. Younger children (aged 5–12) primarily expressed the joy they had from daily conversations with their dying parent. Children aged 13-17 reflected on how they could best make use of these daily small-talks with ill parents (Keeley & Baldwin, 2012). Either way, such daily socialisation with parents provided children with a sense of safety and assurance that their family relationships were still significant (Keeley & Baldwin, 2012; Keeley & Generous, 2013). Children were often aware of the limited time left to live, thus they considered ‘hanging out’ with their dying parents as a way to express care and affection (Keeley & Generous, 2013; Sheehan et al., 2016). Buchwald et al. (2012) described that living with critically ill parents put children ‘in death’s waiting room’, where they had to accept the parent’s imminent death. They felt obliged to stay at home, accompanying their dying parent, and to acquire first-hand information about their parent’s illness. Despite children’s wish to support and interact with their parents, studies also showed that some children had strategies of their own choice to distract themselves from the parent’s illness and life situation, e.g., sport activities, going out with peers, listening to music, playing video games, or sleeping (Buchwald et al., 2012; Melcher et al., 2015; Sheehan et al., 2016). Children used distraction as a strategy to take breaks from their parent’s illness, and thus made time and privacy for themselves in daily life (Sheehan et al., 2016). However, not all children were active participants in terms of facing their parent’s illness. Three studies found that teenagers sometimes chose to avoid conversations with both parents about a parent’s illness and imminent death (Melcher et al., 2015; Stephenson et al., 2015; Sveen et al., 2015), partly as a self-protection technique to manage usual life and to face the reality of losing a parent in own pace (Sveen et al., 2015), and partly because of children’s fear of illness and the deterioration of the ill parent’s health (Melcher et al., 2015; Stephenson et al., 2015). Younger children (<15 years) from divorced families went through double losses, as they first experienced parental divorce followed by a parent’s critical illness and death (Marcussen et al., 2020). Parental divorces may have caused changes in children’s prior ways of living, which again was challenged by the parent’s illness. Moreover, these children felt unable to deal with conflicts that arose between the two families, leading them to adopt avoidant attitudes towards talking about the ill parent’s deteriorating conditions (Marcussen et al., 2020).

Many children of all ages strived to support both their parents while adjusting to their changing lives. One study, for example, reported that a 12-year-old child tried to influence her ill parent’s disease by thinking positively, hoping that her parent’s illness could be cured, while another 14-year-old child stayed at home, calculating the probability of the ill parent returning home from hospital (Buchwald et al., 2012). Some teenagers demonstrated their support by taking on a new family responsibility (Melcher et al., 2015; Sheehan et al., 2016; Turner & Almack, 2017). Studies found that some teenagers perceived the ill parent as their top priority. They took on multiple responsibilities around the ill parent’s care, such as coordinating or participating in care appointments/encounters, comforting parents, and protecting both parents from situations that might drain their energy, such as household tasks (Melcher et al., 2015; Turner & Almack, 2017). Meanwhile, several teenagers were concerned about the co-parent’s well-being as they also felt responsible for him/her and their siblings (Melcher et al., 2015; Sheehan et al., 2016). Often, those teenagers assumed new roles and functions, bearing increased responsibility in household tasks, caring for younger siblings and family pets in the hope of reducing the co-parent’s everyday burden (Melcher et al., 2015; Sheehan et al., 2016). Such new roles often contributed to the teenagers’ own personal growth (Melcher et al., 2015). However, taking on more family responsibilities was challenging for teenagers from single-family households and families where the healthy parent was at work, as the teenagers spent a lot of time and energy with the ill parent after school, e.g., cooking meals, accompanying them to hospital, resulting in limited spare time (Sheehan et al., 2016).

#### The Children’s Inner will and the Parents’ Initiatives for Family Communication

Teenagers wanted to be included in conversations with their parents about the parent’s illness and imminent death (Fearnley, 2012; Fearnley & Boland, 2019; Keeley & Generous, 2013; Sheehan et al., 2014; Stephenson et al., 2015; Turner, 2017), reporting various strategies regarding family communication relating to the parent’s illness. Some teenagers found support in searching information about a parent’s illness to manage the uncertainty in their everyday lives, getting information from the ill parent first and turning to the co-parent for further information (Stephenson et al., 2015). Some teenagers participated actively in communication with both parents about the ill parent’s situation (Keeley & Generous, 2013; Turner, 2017). Such conversations helped them to understand the ill parent’s health condition and prepare them for the parent’s imminent death (Fearnley & Boland, 2019; Keeley & Generous, 2013). Other children adopted a pending strategy regarding communication with their parents about the critical illness situation, receiving the information passively without entering into a dialogue with the parents about the situation (Sheehan et al., 2014; Turner, 2017). Several teenagers had a balanced strategy of receiving information, carefully considering the amount and content of information they were ready to hear (Sheehan et al., 2014; Turner, 2017). They also assessed the ill parents’ emotions and avoided asking questions that might upset their ill parent (Turner, 2017). When parents and children agreed about open dialogues and information about the illness situation, their children often adapted well in relation to the parent’s critical illness (Sheehan et al., 2014; Turner, 2017), while parents who hesitated regarding open communication often had conflicts with their children (Stephenson et al., 2015; Turner, 2017). In addition, in situations where ill parents did not wish to talk about the disease or chose to completely hide information from their teenagers, teenagers deployed a strategy by combining reality and imagination. For example, some teenagers interpreted the ill parent’s health condition based on their own understanding and assumed that the ill parents’ life expectancy could be prolonged by treatments (Melcher et al., 2015), or denied that their parent was dying (Sheehan et al., 2014).

Younger children also wished to be informed, even though it could be difficult to understand the consequences of the parent’s illness (Lytje & Dyregrov, 2021; Lytje et al., 2022). Some younger children noticed that something was wrong through the ill parent’s physical changes, such as a low voice or pale face (Lytje et al., 2022). However, some parents evidenced that even though they did not initiate a dialogue about the diagnosis, young children might still acquire information of the parent’s illness by overhearing conversations between parents and/or with other significant relatives (Lytje & Dyregrov, 2021), thus making it difficult to hide the truth from children.

Children of all ages were concerned about their parents' illness and impending death. Some parents described their children’s strategies by assessing their children’s reactions to the ill parent’s diagnosis. Children whose strategy was to stay busy at school or home, and participate in related activities were perceived as coping well and as being unaffected by the situation concerning the ill parents, as viewed from the parents’ perspective (Graungaard et al., 2021). Other parents described that some children had a bodily strategy, presenting psychosomatic symptoms such as sleeping problems, pain (Graungaard et al., 2021), and separation anxiety (Buchwald et al., 2012; Graungaard et al., 2021). In some cases, parents interpreted this type of strategy as an age-specific behavioural problem, rather than as an impact of a parent’s critical illness (Graungaard et al., 2021).

***Different ways of constructing meaning in the face of a forthcoming loss***

#### The Expectation of Family Cohesiveness During Difficult Times

Children reported a need for close family relationships during the course of a parent’s illness (Keeley & Generous, 2013; Melcher et al., 2015; Turner & Almack, 2017). They viewed support from parents as an approach to confirm their relationships, which also served as a source to cope with psychological distress in their everyday lives (Keeley & Generous, 2013; Turner & Almack, 2017). Teenagers were fond of the everyday interaction with both parents, even though it consisted of simply spending time together (Keeley & Generous, 2013; Melcher et al., 2015). Meanwhile, they were also interested in conversations initiated by the ill parent, sharing personal life stories or giving advice regarding future challenges (Keeley & Generous, 2013). These interactions created a closeness that was viewed as a positive experience from the teenagers’ perspective, through which they gained a sense of togetherness. This helped them prepare for the loss of a parent and minimised loneliness during the process (Keeley & Generous, 2013; Melcher et al., 2015; Turner & Almack, 2017). Teenagers mentioned that they expected the co-parents to ‘be present’ at home in their everyday life, providing both the teenagers and the ill parent with emotional and practical support. They assessed their co-parents as morally right or wrong in relation to their physical presence or absence (Turner & Almack, 2017).

Certain family interactions, either purposely initiated by parents or through everyday routines, had become a family ritual during the course of a parent’s illness. For example, two young children (aged <10) described their ill parents often playing with them, and children considered such interactions between them and their ill parents as fun. They used it as a buffer to deal with negative emotions accompanying the parent’s dying process (Keeley & Baldwin, 2012). Some children, of all ages, experienced their ill parent’s engagement in them as a shared activity, which was a strategy for those children to maintain the bonding with the ill parent prior to, and even after the parent’s death (Keeley & Baldwin, 2012; Turner, 2020).

#### Meaning-Making of a Parent’s Illness and Death

Some children exerted themselves to make sense of the parent’s illness (Melcher et al., 2015; Sveen et al., 2015; Turner, 2020). Teenagers comprehended the parent’s illness by assessing the information they received, observing, and comparing the ill parent’s health condition from day to day (Melcher et al., 2015). In this way, teenagers recognised the changes in the ill parent’s health and adjusted their strategies to live with or support the ill parent in the present moment (Melcher et al., 2015). It could be difficult for teenagers to find strategies to deal with their ill parent’s suffering (Sveen et al., 2015). Some teenagers reasoned that death would be a relief from suffering for their ill parent (Sveen et al., 2015). Some teenagers described how they made sense of their parent’s illness and imminent death by imagining their own future plans, as they gained a sense of future purpose by planning their own life (Turner, 2020). These teenagers realised the future absence of their parents, allowing them to cherish their everyday lives during the parent’s illness course (Turner, 2020). The future plans were often linked to their ill parent’s desires, as they planned to fulfil their ill parent’s wishes, e.g., go to university, being able to live independently. The ill parent’s support of their intended plans enabled children to believe that these were the right priorities. In addition, some children imagined their lives and future without the ill parent during the illness course, as a way to prepare themselves to understand and accept the foreseeable changes in their own lives (Turner, 2020).

#### The Need for Support Outside the Family

Children expressed a need to talk to an ‘outsider’ about the parent’s illness and life changes. They described a variety of interactions and strategies when being offered support from external networks. Some teenagers looked for support from other relatives or neighbours (Keeley & Generous, 2013; Melcher et al., 2015), or approached school therapists (Sveen et al., 2015) to talk about the parent’s illness and impending death. Teenagers described this strategy as allowing them to freely express their own emotions associated with the dying parent’s illness (Keeley & Generous, 2013; Sveen et al., 2015). Some teenagers described that being accompanied by family pets also provided comfort to cope with distress related to the ill parent’s diagnosis (Melcher et al., 2015). The school environments served as another important platform for supporting children in their everyday life. Teenagers mentioned the strategy of preserving their relationship with teachers and/or peers as helpful in keeping up with their studies and everyday lives (Melcher et al., 2015; Sheehan et al., 2016). Some teenagers expressed that peers supported them emotionally when talking about parental illness, while other teenagers used peers as a pause from the parent’s illness, allowing them to feel that life continued as usual (Sveen et al., 2015). The support from peers continued as a crucial strategy for children even after the parent’s death (Sheehan et al., 2016; Sveen et al., 2015). Children viewed their school life as an independent world from their parent’s illness, which provided a possibility to maintain ‘normality’ in their everyday life (Buchwald et al., 2012; Melcher et al., 2015; Sheehan et al., 2016).

Some teenagers established relationships with healthcare professionals as teenagers wished to acquire information about their parent’s illness (Fearnley & Boland, 2019; Mayo, 2016). However, only a few teenagers got emotional support from healthcare professionals (Mayo, 2016; Sheehan et al., 2014). In general, they received limited assistance from professionals, as the main target of such support was mainly their ill parent (Marcussen et al., 2020; Mayo, 2016; Turner & Almack, 2017). Often, teenagers did not know how to establish contact with healthcare professionals (Mayo, 2016), or missed the opportunities to talk with healthcare professionals, due to the restricted visiting hours at hospitals or the healthcare professional’s limited availability (Fearnley & Boland, 2019; Mayo, 2016). Young children and teenagers reported not being included by healthcare professionals in conversations regarding parent’s illness and imminent death (Fearnley & Boland, 2019; Keeley & Generous, 2013). The children could be unaware of the ill parent’s actual health condition and feel unprepared to deal with the loss of a parent, sometimes feeling anger when the ill parent passed away (Fearnley & Boland, 2019).

## Discussion

The study systematically reviewed qualitative studies on children’s strategies and (inter)actions in their everyday life when living with critically ill parents facing an imminent death both from children’s and parent’s perspectives. Maintaining everyday living while constructing the meaning of forthcoming loss were inextricably linked in children’s daily life when living with critically ill parents. The study shows that children display a variety of ways of acting and interacting and many strategies to handle daily life with an ill parent, ranging from actively supporting and communicating with their healthy and ill parents to distracting themselves and avoiding talking about parental illness, and acknowledging/claiming the healthy parents. Children have strategies to take a break from the parent’s illness situation, such as participating in spare time activities, being together with peers, retreating, and sleeping. Children also use imagination as a way to envision a forthcoming life without a parent and to incorporate thoughts about the present and the future. Some children try to make sense of the situation and let the ill parent’s life stories and wishes guide them in their future plans. Furthermore, some children display bodily strategies, presenting psychosomatic symptoms. Meanwhile, children expect and/or look for support from families as well as from external networks including peers, adult friends, and professionals, in order to make sense of the parent’s imminent death, and how it would affect them and their future. The discussion focuses on two main findings. Firstly, we discuss how children have different strategies to handle their everyday life, even when living with a critically ill and dying parent, which points out that they are not ‘particularly vulnerable’ per se but that they are in a vulnerable situation. Secondly, we discuss the inclusion of children in critically ill parent’s situation and the significance of support for these children. Finally, we discuss the strengths and limitations of the current study.

The current results show that children of all ages were able to develop various strategies to maintain their everyday life at school and at home. They interact with peers and adults, including their parents, to achieve a well-functioning everyday life with respect to new family dynamics. Children’s desire to make their own choices and exert autonomy, as seen in the current results, was also supported by other studies (Marshall et al., 2021; Wray et al., 2022). Although the included studies covered children from a wide range of age groups, a majority of them were teenagers, which should be taken into consideration in terms of the results’ interpretation. Many health policies and research regard children as a ‘particularly vulnerable group’ of the population, calling for an increased attention to children in difficult life situations (Bagattini, 2019; United Nations, n.d.). Obviously, children are neither more nor less vulnerable than other people in populations, and most children are capable and competent to make their own contributions to their daily life, even during difficult life situations (Bagattini, 2019; Skovdal et al., 2009). According to the United Nations (1989) ‘Convention on the Rights of the Child’, children have the right to participate in matters concerning their own lives and make their own decisions. Payler, 2020 shows that children desire to maintain an everyday life to be ‘as usual as possible’ both at home and at school, despite their parent’s life-limiting disease (Payler, 2020). In different ways, most children are life-capable, also in vulnerable situations. They are able to handle play, pleasure, sadness, grief, and changes in life, with varied time for adaptation and in different ways depending on age and personality. Lives, also children’s lives, consist of elements standing alongside each other or following each other, without necessarily being related (Bourdieu, 1995; Järvinen, 2000). In the current study, children showed a need for upholding an everyday life ‘as usual’ despite a difficult life situation, as a way to cope and as a way to look into the future. The need for ‘breaks’ from grieving, responsibilities, and thoughts about the ill parent, is a resource for the children to keep up with their everyday life, even in vulnerable life situations. One can say that it is a human condition to have to deal with changes in life, where losing a parent can be considered as a significant and life changing circumstance for a child. However, this does not only apply to children who lose a seriously ill parent; this also applies, for example, to children whose parents divorce or separate, and children whose parents starve to death due to food shortages, or die in natural disasters or war (Lawson et al., 2017; Ratnamohan et al., 2018).

Further, the current study shows that many children of all ages sought information in relation to their parent’s death, using varying strategies. Younger children sensed their parent’s critical illness and imminent death based on obvious physiological facts (e.g., paleness). Teenagers formed their understanding by engaging in conversations with, e.g., their parents and asking questions about the illness and death, and actively participating in daily family routines. This is in line with a previous study showing that children often incorporate different beliefs to form their understandings of death, where the understanding of death influences the response to a parent’s death (Menendez et al., 2020). Furthermore, the current study shows that children wanted to be included in conversations about the parent’s critical illness and death to varying degrees. The importance of communication was highlighted, similarly to the results of Matinceková et al.'s (2020) study, showing that adults who lost a parent in childhood find that open dialogues about parents’ death facilitates their coping abilities and strategies, also in adulthood. Skovdal et al. (2009) find that many children are able to utilise available social resources to cope with the challenges related to parents in poor health condition. Rather than seeing themselves as victims, many children view caregiving experiences as a contribution to their personal growth (Skovdal et al., 2009). Children’s experiences of having a critically ill parent can be regarded as a socialisation to later cope with death in life. The loss of a parent is a saddening event. It can also contribute to a socialisation about death, supporting the children’s strategies with future death-related events (Matinceková et al., 2020). The current study emphasises the potential benefit of communication and activities with peers, parents and/or other adults that support children’s coping strategies regarding having a critically ill and dying parent. This points to a distancing from the medicalisation of children who are to lose/have lost a parent. Such a trend can be seen in the medical field in modern western societies, where vulnerable life situations are often considered as pathological events, to which the answer is treatment, often in the form of psychological interventions aimed at preventing future pathologies of any kind in children (Hagström, 2021; Hawkins, 2021). Being sad and confused may be considered as a healthy (re)action, when being placed in a chaotic situation without influence on its outcome and when trying to re-establish a new order for a new ‘usual’ life during the process of the parental illness course and the subsequent life without one parent. The findings pointed to children being life-capable, also in vulnerable and difficult life situations. The current study calls for future research about health professionals’ and other adults’ awareness of children’s different strategies, of the adults’ capacity to meet the children where they are, respecting their needs and boundaries, and how they support/can support the children’s own coping strategies. The current study points to the necessity of developing children-led support in the future, by listening to, acknowledging, and taking the children’s voices, creativity, experiences, and resources, as starting points to develop adequate support for children in vulnerable life situations such as having a critically ill and dying parent (Fine, 2016; Lindquist-Grantz & Abraczinskas, 2020; Suleiman et al., 2021).

The current review has strengths and limitations. The synthesis of qualitative studies can improve the understanding of experiences or preferences of specific groups (Noyes et al., 2022), here identifying children’s strategies and interactions in everyday life when a parent is critically ill and dying. The selected electronic databases and the construction of the search strings were discussed with and supported by a university librarian, which has strengthened the accuracy of identification of relevant studies. The review was carried out following a predefined research question, search strategies, and analytical methods, which were discussed and agreed upon by all authors throughout the process. The review team consisted of two experienced researchers and two newcomers in the academic field, who collaborated throughout the data collection and analytical processes, strengthening the study’s rigour and credibility. However, this systematic review has several limitations., e.g., studies published in languages other than English were not included. Therefore, there is a risk that the current review has excluded studies of relevance for the topic from the results, limiting their transferability to other contexts. This is also limited by the included study’s origins, which were limited to four countries with westernised traditions only. In addition, the findings were based on the results from studies of varying quality, which also may influence current findings’ trustworthiness. The studies included in the current review were conducted in only four countries, which awakens curiosity since children living with seriously ill and dying parents is a global issue. This calls for further research in additional countries, to make the children’s voices, challenges and resources visible.

## Conclusion

The study showed that children of all ages were capable of developing various strategies to handle their everyday life when living with a critically ill and dying parent, in this case indicating that children are not vulnerable per se but they are in a vulnerable situation. This does not preclude that children, like all individuals, can be vulnerable and in a vulnerable situation. The study recognised that children were active participants in their everyday life during the course of a parent’s illness. Children desired to make independent choices and showed a need for upholding an everyday life ‘as usual’ despite a difficult and vulnerable life situation. The review showed that children searched for information about their parent’s illness and imminent death, and their strategies varied according to their ages. Inclusion, openness and communication with parents, school teachers, peers and other significant persons about the parent’s illness and imminent death, and taking children’s age and needs into consideration, were important for the children to face the situation with a critically ill and dying parent. Further, such experiences with death can help develop children’s coping ability and strategies in life and relating to future deaths. In sum, the findings pointed to most children living with a critically ill and dying parent being life-capable, also in vulnerable and difficult life situations. This calls for the necessity of developing children-led support in the future, by listening to, acknowledging, and taking the children’s voices, creativity, experiences, and resources, as starting points to develop adequate support for children in vulnerable life situations such as having a critically ill and dying parent. Further research about children’s strategies for the re-construction of their everyday lives in and after being in vulnerable situations such as bereavements are needed, as are studies taking into consideration factors such as the families’ social position, the gender and age of children and parents, to better understand the complexities of children’s experiences, challenges, and resources in vulnerable life situations.
